# Identifying Responders to Transcatheter Interventions for Secondary Mitral Regurgitation

**DOI:** 10.1007/s11936-025-01095-1

**Published:** 2025-07-31

**Authors:** Diane Rizkallah, Carl Ammoury, Elio Haroun, Joseph El Roumi, Ben Alencherry, Rhonda Miyasaka, Amar Krishnaswamy, Samir Kapadia, Serge Harb, Deborah Kwon

**Affiliations:** Heart, Vascular and Thoracic Institute, Cleveland Clinic, Cleveland, OH USA

**Keywords:** Secondary mitral regurgitation, Echocardiography, Computerized tomography, Cardiac magnetic resonance, Risk stratification

## Abstract

**Purpose of Review:**

This article offers a comprehensive review of the literature on imaging predictors of response to transcatheter interventions for secondary mitral regurgitation (SMR).

**Recent Findings:**

Recent advancements in percutaneous valve therapies have broadened treatment options for high-risk patients with SMR. While recent studies have demonstrated significant reduction in all-cause mortality and hospitalizations with transcatheter edge-to-edge repair (TEER), significant controversy remains. This review highlights the central role of multi-modality imaging to guide therapeutic decision making for optimal treatment response and long-term outcomes.

**Summary:**

Several echocardiographic, computerized tomography (CT) and cardiac magnetic resonance (CMR) imaging parameters are central to identifying optimal conditions for transcatheter therapies. Imaging features have become key predictors of outcomes following transcatheter interventions for secondary mitral regurgitation and provide important risk stratification to guide therapeutic decision making and shared decision making.

## Opinion Statement

Advanced multimodality imaging is essential for appropriately selecting patients with secondary mitral regurgitation (SMR) who are most likely to experience survival and symptom benefit following transcatheter mitral interventions. While guideline directed medical therapy is the cornerstone therapeutic recommendation for all patients with secondary mitral regurgitation, comprehensive evaluation with multimodality imaging enables a multi-disciplinary structural and imaging team of experts to accurately identify which patients are most likely to further benefit from percutaneous mitral valve interventions. Dedicated evaluation of mitral valve geometry, myocardial chamber size and function, and composite risk scores enable a more personalized approach to therapeutic and shared decision making. Echocardiographic evaluation, including transesophageal echocardiography is the most widely utilized and most available technique for determination of the severity of SMR and basic mitral valve geometry. Cardiac CT provides augmented assessment of mitral valve anatomy in regard to mitral valve calcification and structural relationship to the left ventricular outflow tract. Cardiac MRI enables important gold standard quantitative assessment of chamber size, cardiac function/mechanics, and tissue characterization to determine likelihood of myocardial functional recovery following transcatheter interventions. Ongoing multimodality imaging research will continue to provide more precise patient selection criteria to derive the most accurate and robust selection criteria to more optimally direct therapeutic management decisions.

## Introduction

Secondary mitral regurgitation (SMR) results from adverse left atrioventricular remodeling and dysfunction in the setting of ischemic and nonischemic cardiomyopathy [1]. It is the most common and untreated form of mitral regurgitation (MR), and its prevalence is on the rise with an expected increase to 4 million cases by 2030 [2]. It affects 50% of patients after myocardial infarction [3] and up to 50% of patients with heart failure [4]. Consequently, SMR is a leading cause of morbidity and mortality in heart failure patients and increases the rate of rehospitalizations, thus impacting healthcare systems around the world [5].

Multiple percutaneous valve therapies and innovations have been rapidly evolving, providing high risk patients with options for intervention that did not previously exist. However, currently MitraClip is the only FDA approved percutaneous therapy, based on the COAPT (Cardiovascular Outcomes Assessment of the MitraClip Percutaneous Therapy for Heart failure patients with Functional Mitral Regurgitation) trial, which demonstrated significant reduction in all-cause mortality and recurrent hospitalization compared to GDMT [6]. However, conflicting results of the MITRA-FR (Multicenter Study of Percutaneous Mitral Valve Repair MitraClip Device in Patients With Severe Secondary Mitral Regurgitation) trial demonstrated no significant improvement in outcomes compared to standard medical therapy [7]. Safety, applicability, and effectiveness of MitraClip was further confirmed in the real-world COAPT Post Approval Study (COAPT-PAS), which also higlighted the importance of optimal patient selection criteria to achieve optimal survival benefit [8]. More recently, the MATTERHORN trial demonstrated non-inferiority of mitral valve transcatheter edge-to-edge repair (TEER) compared to surgical intervention in patients with secondary MR at 1 year, with more favorable safety profile associated with M-TEER, compared to surgical intervention (*p* < 0.001) [9]. Moreover, the Randomized Investigation of the MitraClip Device in Heart Failure: 2nd Trial in Patients With Clinically Significant Functional Mitral Regurgitation (RESHAPE-HR2) study demonstrated that the addition of mitral valve transcatheter edge-to-edge repair (TEER) to GDMT significantly improved functional status and symptoms while reducing the risk of heart failure-related hospitalization. However, significant controversy surrounds the study design and interpretability of the RESHAPE-HR2 study findings. Thus, questions still remain regarding optimal patient selection criteria and determination of predictors of optimal response following transcatheter mitral valve therapy [10–12].

## Defining Procedural Success

Cardiac imaging is central to the assessment and management of valvular heart disease. Echocardiography is the main imaging modality used in the evaluation of the mitral valve (MV), due to its wide accessibility. It allows the examination of MV anatomy and function and investigates the etiology of abnormalities. Doppler echocardiography offers important hemodynamic information about the valve, such as mean gradients and regurgitant volumes. Transesophageal echocardiography (TEE) with three-dimensional imaging is required for a more detailed view of the diseased MV anatomy and is essential for surgical vs. interventional planning, as well as intra-procedural guidance [13–15].

Procedural success in patients undergoing TEER can be defined as a reduction in MR severity from severe to moderate regurgitation (2+) or less, for both primary and secondary MR [16, 17]. Another metric of success is reduction in MR by at least one grade in the absence of significant mitral stenosis. This definition was derived from data demonstrating significantly lower rate of adverse events, namely all-cause mortality, cardiovascular death, heart failure hospitalization at 2-years follow-up, as well as improvement in NYHA class one year after TEER for secondary MR [17]. Conversely, Adamo et al,.highlighted the importance of achieving optimal outcomes after TEER, defined as no or mild MR (0/1+), as absent-mild MR was associated with a lower risk of significant MR recurrence compared to patients with moderate MR (2+). Predictors of optimal outcomes were baseline moderate-to-severe MR (3+) rather than severe MR (4+), left ventricular end-systolic diameter (LVESD) < 70 mm at baseline, and TEER performed in a high-volume center performing more than 20 procedures per year. Additionally, patients with severely dilated ventricles at baseline with an LVESD > 70 mm demonstrated significantly decreased likelihood of reverse remodeling after MV intervention [18]. Interestingly, post-hoc analysis of the COAPT trial demonstrated that although LV reverse remodeling assessed by echocardiography at 6 months was associated with improved 2-year outcomes, extent of remodeling did not appear to be affected by TEER or the extent of residual MR [19]. Postulated mechanisms of survival benefit may be that TEER prevents further progressive adverse remodeling and/or worsening MR. However, it is important to note that this study demonstrated heterogeneity in LV remodeling response as assessed by echocardiography, thus the limitations of echocardiography to accurately assess change in LV remodeling in patients with significant LV dilation may have impacted the results of this study [20, 21]. Thus, larger cohort studies and/or studies utilizing CMR defined LV remodeling are needed for more definitive determination of LV remodeling and potential mechanisms of heterogeneity of LV remodeling response following TEER. Conversely, another post-hoc COAPT analysis demonstrated improved event free survival in both TEER and GDMT study groups for patients who demonstrated improvement in LA reservoir strain by 15% at 6-month follow-up. Notably, TEER patients with improved LA strain demonstrated the most optimal survival benefit, compared to patients receiving GDMT [22].

### Mitral Valve Geometry and Regurgitation Severity Assessment

**Fig. 1:**
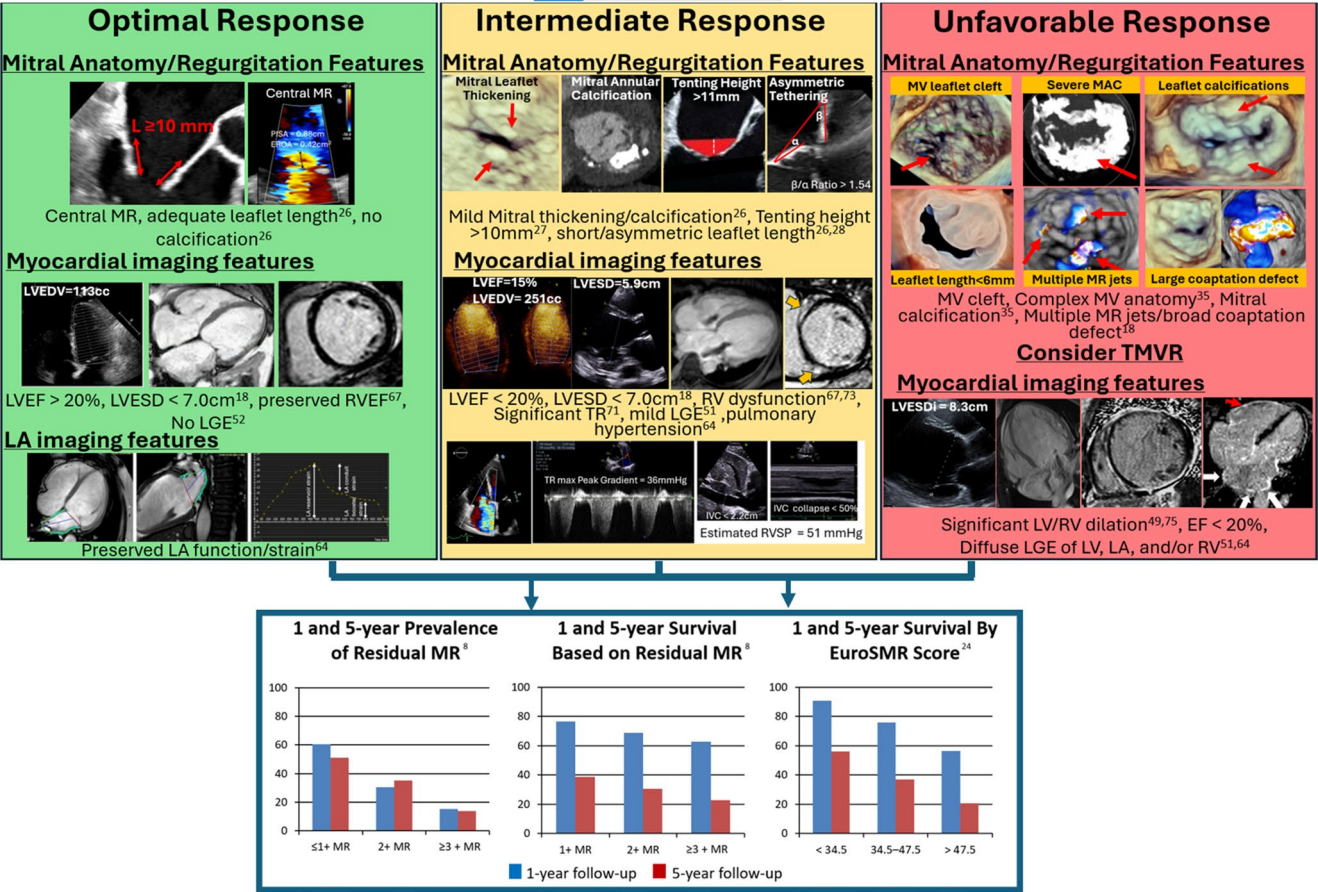
Predictors of transcatheter edge-to-edge repair response and impact on outcomes

Imaging predictors of post-TEER procedural success have emerged in recent studies and integrating these preprocedural measurements into multi-disciplinary discussions have the potential to optimize patient selection for TEER. Less than moderate residual MR has been associated with increased long-term and short-term survival whereas low baseline preprocedural LVEF, particularly in the presence of significant systolic impairment, has been associated with increased mortality [23, 24]. Moreover, several studies have assessed the prognostic value of MV morphologic features in response to M-TEER. Focal origin of SMR has been associated with higher procedural success. Therefore, bicommissural MR resulting in regurgitant jets at both the posteromedial and anterolateral commissures of the MV typically excludes patients from TEER candidacy. Increased LV dilation out of proportion to the size of the MV annulus suggests the presence of annular-ventricular mismatch, increased cardiac work, and more significant myopathic processes, leading to systolic dysfunction and progressive heart failure if not appropriately managed. Therefore, a larger end-systolic 2D-anteroposterior diameter (2D-APd) of the mitral annulus, and a smaller MV area (MVA) to LVEDV ratio (MVA/LVEDV) at baseline have all been found to be significant predictors of suboptimal residual MR after TEER [25]. In addition to a larger MV annular size (> 140 cm²), loss of annular planarity is associated with suboptimal procedural outcomes. Ideally, leaflets should be noncalcified and nontethered, with a length ≥ 10 mm allowing adequate coaptation, while a tenting height > 11 mm carries a worse prognosis [26, 27]. Specifically, a posterior leaflet length < 10 mm is associated with lesser procedural success and increased mortality and morbidity [27]. Asymmetric tethering, which is defined as either a posterior to anterior MV leaflet angle ratio ≥ 1.54 and/or a medial to lateral MV tenting area ratio ≥ 1.49, is associated with 2-year mortality following M-TEER [28]. Calcification of either the leaflets or the mitral annulus, and/or < 5 mm of leaflet available for grasp, also predicts suboptimal procedural outcomes [26]. Finally, a small mitral valve area, particularly below 4 cm^2^ is also associated with suboptimal post-procedural outcomes [26, 27]. These cutoffs and criteria for MV geometry/morphology and MR characteristics are grouped into 3 classifications, ranging from favorable (green zone), to complex (yellow one) and lastly to prohibitive (red zone) to guide decision making regarding TEER vs. TMVR referral [29]. Figure 1 summarizes the concepts discussed in this review and categorizes them in this framework of green/yellow/red zones.

While cardiac CT is not typically involved in the initial assessment of secondary regurgitant valvular lesions, it constitutes a cornerstone of pre-procedural planning prior to transcatheter intervention despite its limited temporal resolution and limited capabilities for tissue characterization. Cardiac CT provides accurate anatomical assessment of the mitral valve and associated calcification [30]. It also provides superior spatial resolution allowing better characterization and delineation of the mitral valve annulus anatomy, as well as LV and LA dimensions, despite suboptimal regurgitation quantification and tissue characterization [31–34]. In fact, more than half of patients referred for TMVR are eventually deterred from undergoing the procedure due to CT-identified contraindications [30]. Careful pre-procedural planning with CT is required to identify patients at risk for post-procedural LV outflow tract (LVOT) obstruction [30]. LVOT obstruction is defined as an increase of at least 10 mmHg in the LVOT gradient and is as associated with worse outcomes including death and technical failure [35]. LVOT obstruction after TMVR is more likely in the presence of certain MV anatomical features, such as an anterior mitral leaflet length (AML) > 2.5 cm, anterior leaflet area > 8 cm2 [26], or a narrow aortomedial angle ≤ 110–115° [26, 36].

While echocardiography remains the first-line imaging modality for MR assessment, CMR has acquired importance in evaluating MR, particularly when echocardiography is suboptimal, or assessment of underlying myocardial substrate is pertinent. Furthermore, CMR is less prone to measurement errors with increasing LV size and volume [37]. and is considered the gold standard for evaluating cardiac chamber size and volume [38]. CMR provides unique, gold-standard tissue characterization and fibrosis assessment using late gadolinium enhancement (LGE) imaging [37].

Significant controversy regarding the optimal threshold to define SMR, and CMR also reclassifies a significant number of severe MR cases defined by echocardiography [39, 40] which has been shown to better predict left ventricular remodeling following intervention [39, 41]. Prior studies have demonstrated that CMR-specific thresholds for SMR severity demonstrated prognostic significance [42–44]. Moreover, 4D-flow CMR is an exciting technique that provides the potential for further improving the accuracy of regurgitant volume and fraction calculation. Further studies are needed to validate the incremental clinical value over 2D flow quantification of MV regurgitation [45], 4D-flow is a promising technique that could provide further insights. Nevertheless, CMR is limited by its suboptimal characterization of MV anatomy and calcification, as well as its vulnerability to artifacts in patients with significant arrhythmia and devices.

#### Left Ventricular Geometry, Function and Tissue Characterization

In recent years, the concept of disproportionate vs. proportionate MR has emerged to explain the discrepancy in outcomes between the MITRA-FR trial and the COAPT and RESHAPE-HF2 trials. Grayburn et al.. classification based on the degree of mitral regurgitation integrated with the quantification of LV end diastolic volume by echo [46]. Initially, proportionate MR was defined as an EROA/LVEDV ratio < 0.14 mm^2^/mL, while disproportionate MR was identified if the ratio was greater than 0.14 [47]. Orban et al. further built on the framework using the EuroSMR registry, and further stratified patients 3 groups: MR-dominant (MD), MR-LV-co-dominant (MLCD), and LV-dominant (LD). MD was defined as EROA/LVEDV ratio ≥ 0.165 cm2 per 100mL of LVEDV, and would be synonymous with the previously classified disproportionate MR. MLCD was defined as EROA/LVEDV range between 0.115-0.165, which is synonymous with proportionate MR. LD was defined as an EROA/LVEDV < 0.115, which would be considered non-severe MR. In this study, two-year mortality after TEER was significantly higher in the LD group compared to the MD and MLCD; however, all patient groups experienced symptomatic improvement, as demonstrated by NYHA classification, 6-minute walking test results, quality of life questionnaires and NT-ProBNP levels [48].

Additionally, LV dysfunction manifested by an LVEF < 40% on echo or a forward SV < 50 mL have been associated with adverse outcomes following TEER. From a structural standpoint, LVESV > 110 mL, or an LVEDV > 216 mL, are also adverse prognostic markers [49].

Myocardial fibrosis (MF) assessed by CMR by late gadolinium enhancement (LGE) provides important insights into myopathic processes. LGE of the lateral and inferior LV walls have been shown to be predictors of MR recurrence post-surgical annuloplasty [50] and severity of LGE was associated with increased adverse outcomes following surgical mitral valve intervention [43]. Similarly, Venu et al. demonstrates that patients with significant LGE, often in the inferolateral wall, experienced adverse outcomes including a NYHA class ≥ III or death within one month following TEER [51]. While patients with limited fibrosis may benefit from interventions, those with significant and widespread fibrosis are less likely to demonstrate significant clinical improvement [52]. Furthermore, T1 mapping is emerging as an additional modality with prognostic significance, as demonstrated by recent studies showing that myocardial fibrosis as determined by T1 mapping was associated with enhances ventricular reverse remodeling following mitral valve surgery [53]. Further studies are needed to determine if LGE and T1mapping can better identify which patients would derive improved outcomes with transcatheter interventions [52].

While cardiac CT is not routinely acquired for TEER planning, cardiac CT provides essential anatomical evaluation for patients undergoing TMVR evaluation to determine the risk of LVOT obstruction following TMVR, [54]. A post-procedural LVOT, referred to as the neo-LVOT, of 1.7 cm2 or less signifies a patient is at high risk for post-TMVR LVOT obstruction and should refrain from undergoing the intervention [30, 35, 55]. Patients with valve-in-MAC are at highest risk for LVOT obstruction, followed by valve in ring and valve in valve patients [30, 35]. An additional high-risk feature predictive of LVOT obstruction is basal septal hypertrophy, particularly when the thickness exceeds 14 mm [56]. Furthermore, multiphasic ECG-gated cine cardiac CTA demonstrated that thinned LV segments was associated with less significant reverse remodeling at 1-month post-procedure, and higher LVEDVi and RVEDVi at baseline was associated with greater decreases in LVEDVi and RVEDVi respectively [57]. Finally, in recent years, the potential to study tissue characterization on cardiac CT has been explored with extracellular volume quantification, late iodine enhancement and strain analysis [58]. While these techniques are not commonly used in clinical practice, due to additional radiation requirements, CT may be able to provide similar insights when MRI cannot be performed.

#### Assessment of Left and Right Atrial Geometry and Function

Although the evaluation of cardiac remodeling in the setting of SMR has conventionally prioritized assessment of left ventricular size and function, the role of atrial parameters in post-TEER prognostication is increasingly recognized. Atrial secondary mitral regurgitation has emerged as a distinct category of SMR in recent years. In such cases which have been linked to heart failure with preserved ejection fraction and atrial fibrillation, left atrial dilation leads to mitral annular enlargement in the absence of LV function impairment [59]. Atrial SMR (ASMR) has also been suggested to be an independent predictor of mortality [60].

Emerging evidence has shown that a decrease in left atrial pressure (LAP) following M-TEER is associated with better outcomes. A post-procedural LAP ≤ 15 mmHg, as measured by echocardiography, constitutes an optimal hemodynamic response to TEER, and is associated with a significantly lower all-cause mortality and heart failure admission at one-year follow-up [61, 62]. Samimi et al. have shown that predictors of higher LAP, such as atrial fibrillation (AF), are therefore also predictors of suboptimal outcome. Higher baseline LAP, which is a marker of diastolic dysfunction, and a postprocedural mitral gradient of < 5 mmHg were also associated with worse outcomes. Measurement of LAP may therefore allow more effective selection of patients on one hand and triaging of those who may not benefit from the procedure on the other hand. Second, acknowledging the importance of LAP may also offer additional insights, especially for patients whose MR grade appears borderline or uncertain on echocardiography [61].

 CMR also allows gold-standard quantification of LAVi, assessement of LA function, and a potential ability to estimate PCWP using LA volume and LV mass on CMR has also been suggested [63]. Cardiac CT can also provide elevated LA pressures by assessing the presence of the pulmonary vein engorgement [64].

Furthermore, structural changes in atrial dimensions and volumes are usually reflective of changes occurring in the ventricles. Left atrial volume index (LAVi) provides insights into chronically elevated LAP, and has been termed the “HbA1c” of the heart [65], reflecting its increasing recognition as an important predictor of overall cardiac function. Moreover, right atrial volume indexed to BSA (RAVi) has been found to be equally important as LAVi in predicting response to TEER in both degenerative and secondary mitral regurgitation, as demonstrated by Miller et al. Patients were divided into four groups according to RAVi and LAVi above or below the median (36.8 ml/m² and 55.4 ml/m², respectively), measured by echocardiography. Interestingly, patients with a high RAVi to LAVi ratio, due to high RAVi and low LAVi, had a significant increase in all-cause mortality after TEER as compared to those having a low RAVi to LAVi ratio, due to low RAVi and high LAVi. This finding was due to high RAVi/low LAVi group having higher mean pulmonary artery (mPAP) and pulmonary capillary wedge pressures (PCWP), compared to the low RAVi/high LAVi group. In addition, left atrial dilation and remodeling as evidenced by an increase in LAVi may be protective against left atrial pressure increase, and subsequent mPAP, PCWP, and right sided pressure expansions [66]. Lastly, left atrial function also plays a prognostic role as change in LAEF has been found to be an independent predictor of all-cause mortality following TEER, with the direction of the change correlating inversely to mortality [67].

### Right Ventricular Geometry, Function and Concurrent Tricuspid Regurgitation

Studies in recent years have demonstrated the importance of RV function and size and their associated prognostic implications following transcatheter mitral valve interventions. RV function is typically assessed on echocardiography using tricuspid annular plane systolic excursion (TAPSE), fractional area change, 3D RVEF, or RV global longitudinal strain. Earlier studies have shown that RV dysfunction or failure, as assessed by TAPSE < 15–16 mmHg, is associated with worse post-procedural outcomes and lower likelihood of response to MitraClip placement [49, 68]. More recently, an echocardiographic parameter referred to as RV to pulmonary artery coupling (RV-PA coupling) has emerged as an additional prognostic indicator. RV-PA coupling can be expressed as a ratio of a functional RV metric such as TAPSE or RV longitudinal strain (RVLS) to pulmonary artery pressure (PAP) [69]. Both TAPSE/PASP and RVLS/PASP have been shown to be associated with increased mortality following M-TEER [69]. Furthermore, improvement in post-procedural TAPSE/PASP at short-term follow up compared to baseline was shown to be associated with decreased long-term mortality following M-TEER [70]. Independent predictors of TAPSE/PASP improvement include low post-procedural mitral mean gradient, low TAPSE, high PASP, and tricuspid regurgitation (TR) at least moderate in severity. Patients with known pre-procedural RV dysfunction as demonstrated by TAPSE < 15 mmHg were more likely to experience improvement in their RV-PA ratio when they concomitantly had a higher LVEF and PASP [70]. Furthermore, moderate-to-severe TR was found to be an independent predictor or 3-year all-cause mortality following M-TEER [71]. The presence of pulmonary hypertension as determined by a PAP > 50–60 mmHg has also been found to be associated with worse outcomes following M-TEER. Shamekhi et al. suggested a risk stratification model determined by echocardiographic criteria. Patients at lowest risk where those with severe MR in the absence of right-sided heart failure. Patients at intermediate risk additionally had at least moderate TR in the absence of RV dysfunction. Finally, patients at highest risk were those with overt RV dysfunction as demonstrated by a right ventricular fractional area change < 35% with TAPSE < 17mmHg, or those with right atrial area > 25 cm^2^, or those with RV volume index > 30 mL/m^2^ [72]. Patients with more extensive right-sided dysfunction experienced increased 1-year mortality following M-TEER compared to those with isolated left-sided dysfunction. Similarly, advanced cardiac dysfunction was an independent predictor of mortality at the 1-year mark.

CMR remains the gold-standard for quantification of right-sided cardiac function and volumes. Recent CMR-based studies have shown that RV ejection fraction (RVEF) and RV end-diastolic volume indexed to BSA (RVEDVi) are predictors of 1-year all-cause mortality following M-TEER, even when adjusting for LVEF and LVEDVi [67], and that RVEF ≤ 40% is associated with worse outcomes in patients with NICM and SMR regardless of the severity of SMR and myocardial fibrosis [73], thus emphasizing the importance of integrating quantitative RV assessment with SMR evaluation.

### Risk Scores Predicting Outcomes Following M-TEER

In addition to their role as individual and independent predictors of response following transcatheter mitral valve interventions in the setting of SMR, various imaging parameters have been incorporated into comprehensive clinical risk scores [74]. In 2022, the COAPT score was developed to predict 2-year mortality and heart failure hospitalization risk following M-TEER [75]. It integrated 4 clinical variables and 4 echocardiographic parameters, namely LVEF, LVESD, RVSP and TR. In the same year, the MitraScore was developed by integrating 8 predictors of mortality, including LVEF as determined by echocardiography [76]. The MitraScore was not only predictive of mortality but also heart failure rehospitalization following M-TEER. More recently, the EuroSMR risk score was developed in 2023, integrated 18 clinical and echocardiographic variables, including TAPSE, RA area, sPAP, LVEDV, TR severity, RV mid-diameter, EROA, LAV and LVEF, to predict mortality and clinical improvement 1 and 5 years after M-TEER [77] (Fig. 1). To date, no validated risk score has integrated imaging parameters derived from advanced cardiac imaging modalities.

#### Sex-Based Differences in Transcatheter Interventions for Secondary Mitral Regurgitation

Secondary mitral regurgitation is more prevalent in men, with significant sex associations with the underlying cardiomyopathy phenotype. Women are more likely to have concomitant non-ischemic cardiomyopathy (NICM), whereas the men are more likely to have concomitant ischemic cardiomyopathy (ICM). However, when women develop cardiomyopathy in the setting of ischemic heart disease, they are at higher risk of developing SMR compared with their male counterparts [13, 39]. Furthermore, prior studies have shown that women are likely to present with more advanced disease, with relatively larger LA and LV sizes, and MR volumes compared to their male counterparts [13, 39]. Women have also been shown to have larger EROA/LVEDV ratio [39], which may be thought to suggest a higher prevalence of disproportionate SMR compared to males. However, this classification system is not sex specific, and the proportionate-disproportionate paradigm does not consider that women have smaller ventricles than males, and that a similar EROA/LVEDV ratio in males and females reflects a relatively larger LV in the latter. Hence, despite being classified as having disproportionate MR, females may be more prone to proportionate MR, which is a poorer prognostic indicator [78]. Similarly, available risk scores also fail to account for inherent differences in cardiac dimensions between men and women. Women have been consistently underrepresented in most clinical trials [13, 79]. One prior study demonstrated that women experience more significant reverse remodeling following transcatheter intervention, and that female sex is a predictor of reverse remodeling following TEER [80]. In this sense, female sex may be a predictor of successful response following TEER with similar survival and long-term outcomes [79, 81–83]. However, a study from the Cardiothoracic Surgical Trials Network demonstrated significantly higher adverse outcomes in women with severe ischemic mitral regurgitation undergoing mitral valve surgery, and reduction in heart failure hospitalization in the COAPT trial was less significant in women, compared to men, after the first year following MitraClip therapy [81, 84–86]. Additionally, atrial fibrillation has emerged as a potential predictor of adverse outcomes in females, but not males. As demonstrated by Ausbuettel et al. in their multicenter observational study, atrial fibrillation was observed higher cumulative mortality at the 3-year mark only in females [79]. More dedicated studies are needed to evaluate the presence of sex-specific differential risk prediction based on the current risk scores and imaging predictors to provide optimized patient selection and prognostic criteria for both men and women.

## Conclusions

In conclusion, transcatheter interventions have emerged as an important complimentary therapeutic strategy to GDMT for certain patients with SMR. Several imaging parameters and risk scores have emerged as prominent predictors of response to these transcatheter interventions, thus enabling improved risk stratification and patient selection. While recent studies have demonstrated sex differences in certain outcomes following TEER have, future studies are needed to more definitively ascertain these differences to establish the need for sex-specific predictors, risk scores and guidelines.

## Key References

- Goel K, Lindenfeld J, Makkar R, Naik H, Atmakuri S, Mahoney P, et al. Transcatheter Edge-to-Edge Repair in 5,000 Patients With Secondary Mitral Regurgitation: COAPT Post-Approval Study. J Am Coll Cardiol. 2023;82(13):1281-97.
  - Findings from this study suggest that mitral valve transcatheter edge-to-edge repair (M-TEER) is a safe and effective option in patients with secondary mitral regurgitation.
- Baldus S, Doenst T, Pfister R, Gummert J, Kessler M, Boekstegers P, et al. Transcatheter Repair versus Mitral-Valve Surgery for Secondary Mitral Regurgitation. N Engl J Med. 2024;391(19):1787-98.
  - Findings from this study comparing M-TEER to surgical intervention in patients with secondary mitral regurgitation at 1-year follow-up, highlighted the non-inferiority of M-TEER compared to surgery.
- Gheorghe LL, Mobasseri S, Agricola E, Wang DD, Milla F, Swaans M, et al. Imaging for Native Mitral Valve Surgical and Transcatheter Interventions. JACC Cardiovasc Imaging. 2021;14(1):112-27.
  - Recent advancements in transcatheter therapies for mitral regurgitation require a thorough understanding of mitral valve anatomy, informed by surgical valve repair principles. Integrated imaging plays a crucial role in planning, guiding, and evaluating these procedures. This review offers a targeted update on the intersection between contemporary surgical and transcatheter therapies for mitral regurgitation from an imaging perspective.
- Lim DS, Herrmann HC, Grayburn P, Koulogiannis K, Ailawadi G, Williams M, et al. Consensus Document on Non-Suitability for Transcatheter Mitral Valve Repair by Edge-to-Edge Therapy. Structural Heart. 2021;5(3):227-33.
  - Findings from this study identify cutoffs and criteria for mitral valve geometry, morphology, and regurgitation characteristics. It categorizes patients into three groups: favorable (green zone), complex (yellow zone), and prohibitive (red zone), to help guide clinical decision-making between transcatheter interventions.

## Data Availability

No datasets were generated or analysed during the current study.
